# Endovascular Versus Open Repair for Descending Thoracic Aortic Aneurysm: A Systematic Review of Outcomes in Younger Patients (Under 65 Years)

**DOI:** 10.7759/cureus.96138

**Published:** 2025-11-05

**Authors:** Husam Eldin Abuelgassim Hassan Balila, Mohamed Osman Suliman Basher, Mohamed Abdelmagid Eltahir Hamza, Eman Abubakeralsideeg Diaaldeen Mohamed, Inaam Fadllallah SaidAhmed Othman, Abdelrafi Ali

**Affiliations:** 1 General Surgery, United Lincolnshire Teaching Hospitals NHS trust (Pilgrim Hospital), Boston, GBR; 2 General Surgery, Cork University Hospital, Cork, IRL; 3 Surgery, King Salman Armed Forces Hospital, Tabuk, SAU; 4 General Surgery, Sudan Medical Specialization Board, Khartoum, SDN; 5 Diabetes and Endocrine, South Tees Hospitals NHS Foundation Trust, Middlesbrough, GBR; 6 Accident and Emergency, Epsom and St Helier University Hospitals NHS Trust, London, GBR

**Keywords:** descending thoracic aortic aneurysm, open surgical repair, outcomes, perioperative mortality, reintervention, systematic review, tevar, younger patients

## Abstract

The management of descending thoracic aortic aneurysms (DTAAs) in younger patients (<65 years) presents a unique challenge, balancing the minimally invasive benefits of thoracic endovascular aortic repair (TEVAR) against the potential long-term durability of open surgical repair (open aortic repair (OAR)). While TEVAR is established as a superior option in older, higher-risk cohorts, its outcomes relative to OAR in a younger demographic with longer life expectancy remain unclear. This systematic review aims to compare the perioperative and long-term outcomes of TEVAR versus OAR specifically in younger patients. A systematic literature search was conducted across PubMed/MEDLINE, Scopus, Web of Science, and Embase from 2015 to 2025, following the Preferred Reporting Items for Systematic Reviews and Meta-Analyses (PRISMA) guidelines. Studies were included if they directly compared TEVAR and OAR for DTAA in patients under 65 years. Two reviewers independently performed study selection, data extraction, and risk-of-bias assessment using the Risk Of Bias In Nonrandomized Studies-of Interventions (ROBINS-I) tool. A narrative synthesis was undertaken due to significant clinical heterogeneity. Eight retrospective studies, encompassing 55,621 patients, were included. The evidence consistently demonstrated that TEVAR was associated with superior perioperative outcomes, including significantly lower mortality, reduced stroke rates, shorter hospital stays, and decreased transfusion requirements. However, TEVAR was consistently linked to a substantially higher long-term reintervention rate compared to OAR. The evidence on long-term survival was conflicting, with some studies showing comparable rates and others suggesting a potential survival advantage for TEVAR. For younger patients with DTAA, TEVAR offers a favorable perioperative safety profile but at the cost of significantly reduced long-term durability, necessitating a higher rate of reinterventions. The choice between strategies should be individualized, weighing the short-term benefits of TEVAR against the long-term durability of OAR, with particular caution advised against the elective use of TEVAR in patients with connective tissue disorders.

## Introduction and background

Descending thoracic aortic aneurysm (DTAA) represents a life-threatening condition characterized by progressive dilatation of the thoracic aortic wall, carrying a substantial risk of rupture and mortality if left untreated [1]. Traditionally, open surgical repair has been the standard approach for managing DTAA, offering durable long-term results through direct replacement of the diseased aortic segment [2]. However, open repair is associated with considerable perioperative morbidity and mortality, particularly due to the invasive nature of thoracotomy, the need for aortic cross-clamping, and the potential for spinal cord ischemia, pulmonary complications, and prolonged recovery [3].

Over the past two decades, the advent of thoracic endovascular aortic repair (TEVAR) has revolutionized the management of thoracic aortic diseases. TEVAR offers a minimally invasive alternative by deploying a stent-graft within the aorta via transfemoral access, thereby avoiding thoracotomy and aortic cross-clamping [4]. Numerous studies have demonstrated that TEVAR reduces early morbidity and short-term mortality compared with open repair, especially in elderly or high-risk patients [5]. Consequently, TEVAR has become the preferred modality for the majority of patients with suitable anatomy, as reflected in current vascular surgery guidelines [6].

Nevertheless, concerns remain regarding the long-term durability of TEVAR, particularly in younger patients. Unlike older individuals with limited life expectancy, patients under 65 years may live long enough to experience late device-related complications such as endoleak, stent migration, or need for reintervention [7]. In contrast, open repair, though associated with greater early risk, provides a more definitive anatomical correction with potentially superior long-term freedom from reintervention [8]. The balance between the early safety of TEVAR and the long-term durability of open repair remains a key clinical dilemma in younger, lower-risk populations [9].

Despite growing literature comparing TEVAR and open repair, most existing studies have predominantly focused on older or mixed-age cohorts, with limited evidence specifically addressing younger patients. Age-specific differences in outcomessuch as survival, reintervention rates, and aortic-related complications, are essential for guiding individualized treatment strategies and refining patient selection criteria [10]. Understanding these differences is particularly relevant as younger patients are more likely to have connective tissue disorders, traumatic aortic injuries, or degenerative aneurysms that may influence both procedural outcomes and long-term durability.

Therefore, this systematic review aims to comprehensively compare the outcomes of endovascular versus open surgical repair of DTAAs in younger patients (<65 years). By synthesizing current evidence from recent clinical studies, this review seeks to evaluate perioperative mortality and morbidity, long-term survival, reintervention rates, and device-related complications, thereby providing insights into the optimal management strategy for this distinct patient population.

## Review

Methodology

Protocol and Reporting Framework

This systematic review was conducted in accordance with the Preferred Reporting Items for Systematic Reviews and Meta-Analyses (PRISMA) 2020 guidelines [11]. The review process followed a pre-defined protocol that outlined the research question, eligibility criteria, search strategy, and methods for data extraction and quality assessment. The protocol was developed to ensure transparency, reproducibility, and methodological rigor throughout all stages of the review.

Eligibility Criteria

Studies were included if they directly compared outcomes of endovascular (TEVAR) and open surgical repair of DTAA in younger patients aged under 65 years. Eligible studies included randomized controlled trials (RCTs), cohort studies, and case-control studies published between January 2015 and August 2025. This 10-year timeframe was selected to ensure inclusion of the most recent and methodologically robust evidence reflecting advancements in both endovascular technology and surgical techniques. Studies were required to report at least one of the following outcomes: perioperative mortality, long-term survival, complication rates, reintervention rates, or aortic-related adverse events. Case reports, reviews, editorials, conference abstracts, and studies involving patients with aortic dissections, traumatic aortic injuries, or CTDs without separate subgroup data were excluded.

Information Sources and Search Strategy

A comprehensive literature search was conducted across four major electronic databases: PubMed/MEDLINE, Scopus, Web of Science, and Embase (Elsevier). The search covered all relevant studies published from January 1, 2015, to August 30, 2025. To enhance completeness, additional records were identified through manual searching of reference lists of relevant articles and systematic reviews. The search strategy combined controlled vocabulary terms (e.g., MeSH) and free-text keywords related to “descending thoracic aortic aneurysm,” “endovascular repair,” “TEVAR,” “open repair,” and “younger patients.” The full search strategy was tailored to each database to maximize sensitivity and specificity.

Selection Process

All identified records were imported into EndNote X9 (Clarivate Analytics) reference management software, where duplicates were removed automatically and manually verified to ensure accuracy. Two reviewers independently screened titles and abstracts to identify potentially eligible studies. Full texts of shortlisted articles were then retrieved and assessed against the inclusion and exclusion criteria. Disagreements between reviewers were resolved through discussion or, when necessary, consultation with a third reviewer to reach consensus.

Data Collection Process

Data extraction was performed independently by two reviewers using a standardized data extraction form developed for this review. Extracted information included study characteristics (author, year, country, study design, sample size), patient demographics, procedural details (TEVAR vs open repair (OR)), follow-up duration, and outcomes of interest (mortality, complications, reinterventions, and survival). Where essential data were missing, attempts were made to contact the corresponding authors for clarification. Extracted data were cross-checked for consistency and accuracy before synthesis.

Risk of Bias Assessment

The methodological quality and risk of bias of the included non-randomized studies were assessed using the Risk Of Bias In Non-randomized Studies of Interventions (ROBINS-I) tool [12]. This tool evaluates bias across seven domains, including confounding, participant selection, intervention classification, deviations from intended interventions, missing data, outcome measurement, and selection of the reported result. Each domain was graded as low, moderate, serious, or critical risk of bias, and an overall judgment was derived for each study. Two reviewers independently performed the assessment, and discrepancies were resolved by consensus.

Data Synthesis

Due to substantial heterogeneity across the included studies in terms of study design, patient selection criteria, intervention techniques, follow-up duration, and outcome measures, a meta-analysis was not conducted. The included studies exhibited wide variability in the definitions of outcomes, reporting methods, and adjustment for confounding factors, making quantitative pooling of data inappropriate and potentially misleading. Instead, a narrative synthesis approach was adopted to summarize the key findings, highlight consistent trends, and compare the relative outcomes of endovascular versus OR in younger patients. Results were organized thematically according to short- and long-term outcomes, reintervention rates, and complication profiles.

Reporting

This review adhered to the PRISMA 2020 reporting standards to ensure methodological transparency and completeness [11]. A PRISMA flow diagram was prepared to illustrate the study selection process, including the number of records identified, screened, excluded, and included in the final synthesis.

Results

Study Selection and Characteristics

**Figure 1:**
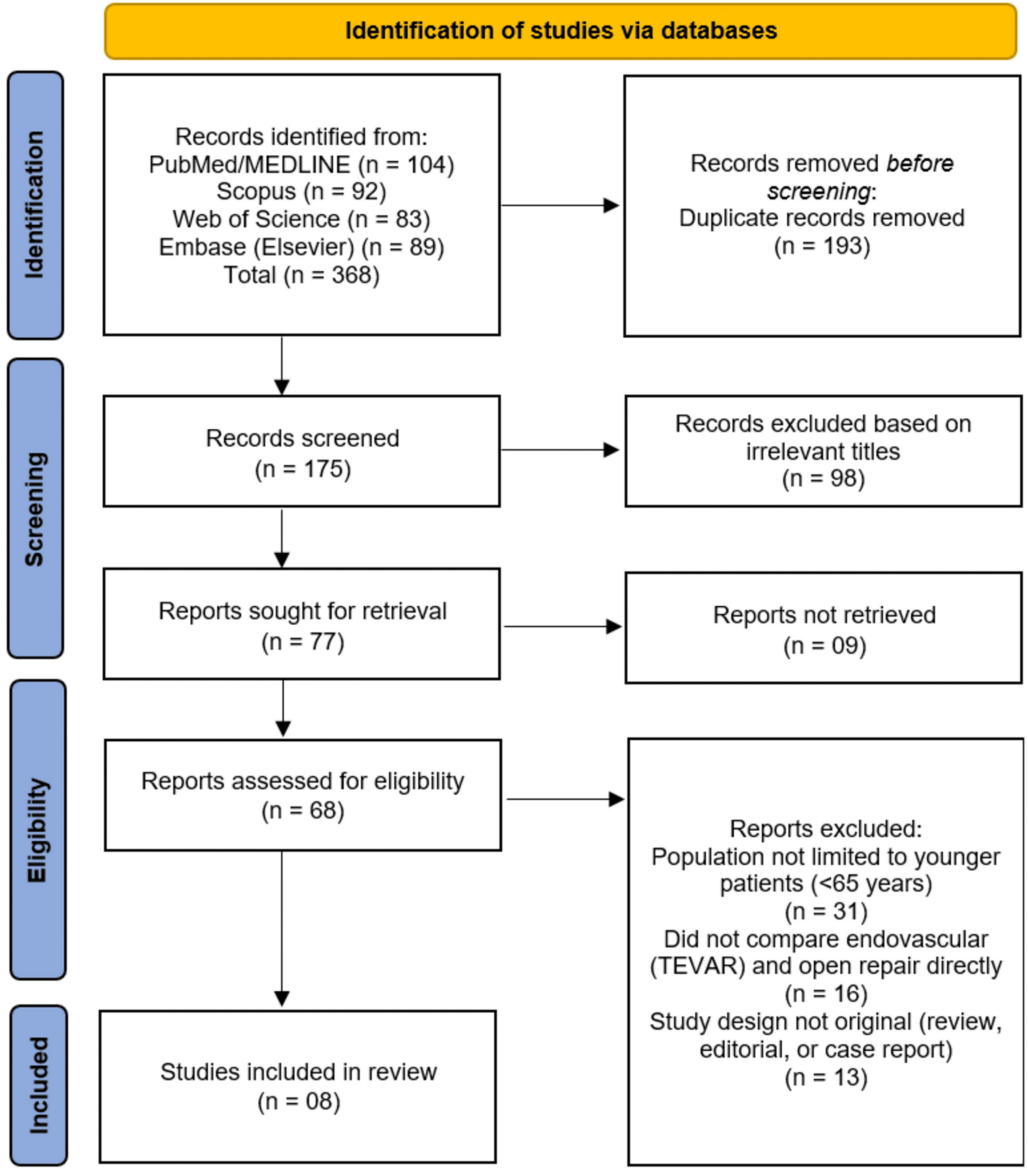
Studies' identification process on the PRISMA flowchart PRISMA:  Preferred Reporting Items for Systematic Reviews and Meta-Analyses

The systematic search across four electronic databases (PubMed/MEDLINE, Scopus, Web of Science, and Embase) initially identified 368 records. After the removal of 193 duplicate records, a total of 175 unique publications were screened by title. This initial screening phase led to the exclusion of 98 records that were deemed irrelevant. The remaining 77 reports were sought for retrieval, of which nine could not be acquired. The full texts of the resulting 68 articles were thoroughly assessed for eligibility. Of these, 31 studies were excluded because the study population was not limited to younger patients (<65 years), 16 were excluded for not providing a direct comparison between TEVAR and OR, and a further 13 were excluded due to an ineligible study design (e.g. review, editorial, or case report). This rigorous process culminated in the inclusion of eight studies [13-20] that met all the pre-defined criteria for this systematic review. The study selection process is detailed in the PRISMA flow diagram (Figure 1).

A total of eight studies [13-20] were included in this systematic review, all of which were retrospective in design, comparing outcomes of TEVAR and open surgical repair (open aortic repair (OAR)) for DTAAs in patients under 65 years of age. The characteristics of these studies are summarized in Table 1. The studies were published between 2017 and 2024 and originated from a diverse range of geographical regions, including the United States [13,19,20], Japan [16,17], China [14], Germany [18], and one global, multicenter investigation [15]. Sample sizes varied considerably, from a single-center study of 63 patients [14] to large-scale national database analyses encompassing over 35,000 patients [17]. The populations studied included patients with intact, ruptured, and CTD-related aneurysms. The follow-up duration ranged from in-hospital outcomes only [16-18,20] to extended periods of up to 15 years [19].

**Table 1:** Characteristics of included studies JCVSD: Japan Cardiovascular Surgery Database; DTAA: descending thoracic aortic aneurysm; rTAA: ruptured thoracic aortic aneurysm; TEVAR: thoracic endovascular aortic repair; rDTAA: ruptured descending thoracic aortic aneurysm; OAR: open aortic repair; ER: endovascular repair; OR: open repair; HAR: hybrid arch repair; NR: non-ruptured

Author (year)	Country/region	Study design	Sample size (n)	Age (mean ± SD/range)	Population/inclusion criteria	Intervention	Comparator	Follow-up duration	Primary outcome(s)
Orelaru et al. (2023) [13]	USA	Retrospective cohort study with propensity score matching	499 total (TEVAR: 278; open repair: 221); 120 matched pairs analyzed	<65 years	Patients with descending thoracic aortic aneurysm who underwent open or endovascular repair between August 1993 and February 2023	TEVAR	Open surgical repair	Up to 10 years (7-year reoperation data, 10-year survival data reported)	Postoperative paralysis, operative mortality, reoperation rate, and midterm survival
Lin et al. (2024) [14]	China	Retrospective	63	41 ± 12 yrs	TAAA after prior TEVAR	Prior TEVAR	Open TAAAR	Up to 5 yrs	Mortality, neurological, renal, pulmonary complications, survival
Muncan et al. (2022) [15]	Global (TriNetX Data Network-multicenter, international)	Retrospective cohort study	Total: 255 (TEVAR = 55; open = 200; matched cohorts: 46 vs. 46)	<65 years	Patients with connective tissue disorders and intact DTAAs	TEVAR	Open surgical repair of intact DTAA	5 years	5-year incidence and hazard of death, re-intervention, aortic dissection, renal failure, stroke, intracranial hemorrhage, paraplegia, and limb ischemia
Yamaguchi et al. (2019) [16]	Japan	National registry-based retrospective comparative study	rDTAA: 983 (ER = 472; OR = 511)	<65 years	Patients with rDTAA admitted to certified teaching hospitals between 2012 and 2015	Endovascular repair	Open surgical repair	In-hospital	In-hospital mortality, hospital stay, functional status at discharge, total medical costs
Shimizu et al. (2019)[17]	Japan	Retrospective database analysis (JCVSD, nationwide)	35,427	<65 years	Patients undergoing aortic repair in 2015-2016	TEVAR	OAR, HAR	NR	Operative mortality, stroke, paraplegia, renal failure
Geisbüsch et al. (2019) [18]	Germany	Nationwide population-based retrospective study	4,969 (TEVAR: 4,057; OAR: 912)	69 ± 12 years	All inpatient cases of ruptured or non-ruptured DTA aneurysm who received TEVAR or OAR between 2005 and 2014	TEVAR (n = 4,057)	Open repair (OAR, n = 912)	In-hospital	In-hospital mortality (primary); organ complications (secondary)
Chiu et al. (2019) [19]	USA	Retro cohort	2,470 / 1,235	<65 years	Intact descending TAA	TEVAR	Open repair	Up to 15 yrs	Mortality, survival, reintervention
Ultee et al.(2017) [20]	USA	Retrospective cohort (national inpatient sample)	12,399 (TEVAR: 1,622; open repair: 2,808; non-operative: 7,969)	<65 years	Patients admitted with rTAA from 1993 to 2012	TEVAR	Open surgical repair	In-hospital	In-hospital mortality; secondary: perioperative complications, length of stay, trends in use over time

Perioperative Mortality

The perioperative mortality outcomes consistently favored TEVAR over OAR across multiple studies. In a large US national inpatient sample, Ultee et al. reported a significantly lower in-hospital mortality for TEVAR compared to OR (22% vs. 33%, p < .001) in patients with rTAA [20]. This finding was corroborated by Yamaguchi et al. in a Japanese nationwide registry, where endovascular repair (ER) for ruptured DTAA was associated with reduced in-hospital mortality compared to OR (22.5% vs. 29.8%, p < 0.001) [16]. Similarly, Geisbüsch et al., in a German population-based study, found TEVAR to be associated with a significantly lower risk of in-hospital mortality for both non-ruptured (3.7% vs. 10.5%) and ruptured (22.3% vs. 42.9%) cases, with a relative risk of 0.31 [18]. Chiu et al. also confirmed that OR was independently associated with higher early mortality (OR: 1.97-3.62, p < 0.001) [19]. In the context of intact aneurysms, Orelaru et al. noted a lower, though not statistically significant, perioperative mortality rate for TEVAR (0.8% vs. 4.2%, p = 0.10) [13].

Mid- to Long-Term Survival

Regarding intermediate and long-term survival, the evidence presents a more nuanced picture. Orelaru et al. found no significant difference in long-term survival between the two groups, with 10-year survival rates of 56% for TEVAR and 58% for OR (p = 0.55) [13]. Conversely, Chiu et al. reported that TEVAR was associated with superior mean survival compared to OR, which had a survival deficit of 209.2 days [19]. In a specific population of patients undergoing open thoracoabdominal aortic aneurysm repair after prior TEVAR, Lin et al. reported an acceptable five-year survival rate of 74.8% ± 4.9% [14].

Neurological Complications

The risk of specific neurological complications varied between the two procedures. Orelaru et al. observed a significantly lower rate of postoperative stroke in the TEVAR group compared to the OR group (2.5% vs. 9.2%, p=0.03), while the rates of spinal cord ischemia (SCI) were identical (2.5% vs. 2.5%) [13]. A Japanese database analysis by Shimizu et al. reported stroke rates of 7.3% for TEVAR and 8.4% for OR, with paraplegia rates ranging from 3.4 to 4.6% for TEVAR and 4.3-8.9% for OAR [17]. Lin et al. reported neurological complication rates of 11.1% and renal/pulmonary complications of 7.9% in their cohort undergoing repair after TEVAR [14].

Reintervention and Long-Term Durability

A key trade-off identified across studies was the higher reintervention rate associated with TEVAR. Orelaru et al. found a significantly higher late reoperation rate in the TEVAR group (16.1% vs. 3.6%, p < 0.001), often implied to be due to endoleaks [13]. This was strongly supported by Muncan et al., who reported a reintervention rate of 27.1% for TEVAR versus 4.8% for OR (p = 0.009) in patients with connective tissue disorders (CTDs) [15]. Chiu et al. also confirmed a higher reintervention hazard for TEVAR (HR 0.40 for OR, indicating OR had a lower hazard) [19]. This suggests that while TEVAR offers superior perioperative safety, OR may also provide greater long-term durability and freedom from reoperation.

Other Perioperative Outcomes

Other perioperative metrics consistently favored TEVAR. Orelaru et al. demonstrated that TEVAR was associated with a significantly shorter hospital length of stay (5 vs. 12 days, p < 0.001) and substantially reduced need for blood products (8% vs. 66%) compared to OR [13]. Yamaguchi et al. also reported a shorter hospital stay for ER (25.5 vs. 32 days) [16]. A summary of these and other clinical and statistical outcomes is provided in Table 2.

**Table 2:** Clinical and statistical outcomes of included studies NR: non-ruptured; R: ruptured; TAAAR: total aortic arch replacement; TEVAR: SCI: spinal cord ischemia

Author (year)	Perioperative mortality (%)	Overall survival (%)	Neurological Complications (spinal cord ischemia/stroke) (%)	Reintervention rate (%)	Length of hospital stay (days)	Mean blood loss (mL)	Follow-up complications (endoleak/graft failure)	p-value/statistical significance	Key findings
Orelaru et al. (2023) [13]	0.8 vs 4.2	56 (10 yr) vs 58 (10 yr)	SCI: 2.5 vs 2.5; stroke: 2.5 vs 9.2	16.1 vs 3.6	5 vs 12	Blood products: 8% vs 66%	Higher late reoperation in TEVAR (endoleak implied)	Stroke = 0.03; LOS < 0.001; reop < 0.001; mortality = 0.10; survival = 0.55	TEVAR had fewer strokes, shorter stay, and less blood loss, but higher late reintervention; survival similar to open repair
Lin et al.(2024) [14]	17.5	74.8 ± 4.9 (5 yrs)	11. /7.9	4.8	NR	NR	Endoleak & false lumen progression (pre-op); none specified post-op	NR	High perioperative risk but acceptable 5-year durability after TAAAR post-TEVAR in young patients (mean age 41 ± 12).
Muncan et al. (2022) [15]	NR	NR	NR	27.1 vs 4.8	NR	NR	NR	0.009	Only re-intervention rate significantly higher in TEVAR; other adverse outcomes numerically higher but NS
Yamaguchi et al. (2019) [16]	ER 22.5 / OR 29.8	NR	NR	NR	ER 25.5/OR 32	NR	NR	<0.001	ER had lower perioperative mortality and shorter hospital stay; similar functional status at discharge; cost lower for ER
Shimizu et al. (2019) [17]	Overall 7.3%; TEVAR lowest	NR	Stroke: OAR 8.4, TEVAR 7.3, HAR 10.1; paraplegia: OAR 4.3-8.9, TEVAR 3.4-4.6, HAR 6.3-10.4	NR	NR	NR	NR	NR	TEVAR shows the lowest mortality and morbidity; OAR lowest stroke for non-dissected/unruptured aorta
Geisbüsch et al., [18] (2019)	TEVAR: 3.7% (nr), 22.3% (r); OAR: 10.5% (nr), 42.9% (r)	NR	NR	NR	NR	NR	NR	RR 0.31 (0.23-0.41)	TEVAR associated with lower perioperative mortality; higher age, rupture, and comorbidities increase mortality; elective TEVAR recommended
Chiu et al., [19] (2019)	TEVAR: lower; Open: higher (OR 1.97–3.62)	TEVAR: superior mean survival; Open: lower (−209.2 days)	NR	TEVAR: higher; Open: lower (HR 0.40)	NR	NR	NR	p < 0.001	Open repair → higher early mortality but lower late reintervention; TEVAR → better mean survival
Ultee et al., [20] (2017)	TEVAR: 22%; Open repair: 33%	NR	NR	NR	NR	NR	NR	Mortality: p < .001; trend analyses: p < .001	TEVAR shows lower perioperative mortality than open repair

The synthesized results indicate that in younger patients (<65 years) with DTAA, TEVAR is associated with superior perioperative outcomes, including lower mortality, fewer strokes, and faster recovery. However, this comes at the cost of a significantly higher long-term reintervention rate. Long-term survival appears comparable between the two strategies, though some evidence suggests a potential late survival advantage for TEVAR.

Risk of Bias Assessment

The methodological quality of the included studies, assessed using the ROBINS-I tool, demonstrated variable risk of bias. The majority of studies were judged to have a low overall risk of bias [13,14,16-18,20]. However, two studies were deemed to have an elevated risk; the study by Muncan et al. [15] was assessed as having a serious overall risk of bias, primarily due to moderate risk in confounding and missing data, while the study by Chiu et al. [19] was also judged as serious, stemming from a serious risk of bias in both confounding and missing data domains. Consequently, the body of evidence is comprised predominantly of studies with a low risk of bias, though the conclusions are tempered by the serious risk identified in two of the eight included studies (Table 3) [15,19].

**Table 3:** Risk of bias assessment using the ROBINS-I tool ROBINS-I: Risk Of Bias In Nonrandomized Studies-of Interventions

Author (year)	D1: confounding	D2: selection of participants	D3: classification of interventions	D4: deviations from interventions	D5: missing data	D6: measurement of outcomes	D7: selection of reported result	Overall risk of bias
Orelaru et al. (2023) [13]	Low	Low	Low	Low	Low	Low	Low	Low
Lin et al. (2024) [14]	Low	Low	Low	Low	Low	Low	Low	Low
Muncan et al. (2022) [15]	Moderate	Moderate	Low	Low	Moderate	Low	Low	Serious
Yamaguchi et al. (2019) [16]	Low	Low	Low	Low	Low	Low	Low	Low
Shimizu et al. (2019) [17]	Low	Low	Low	Low	Low	Low	Low	Low
Geisbüsch et al. (2019) [18]	Low	Low	Low	Low	Low	Low	Low	Low
Chiu et al. (2019) [19]	Serious	Low	Low	Low	Serious	Low	Low	Serious
Ultee et al. (2017) [20]	Low	Low	Low	Low	Low	Low	Low	Low

Discussion

This systematic review synthesizes evidence from eight retrospective studies comparing TEVAR to OAR for DTAAs in the distinct and clinically significant population of patients under 65 years of age. The principal findings indicate a clear trade-off: TEVAR is consistently associated with superior perioperative safety, including lower mortality, reduced stroke rates, and faster recovery, but this initial advantage is counterbalanced by a significantly elevated risk of long-term reintervention. Long-term survival, however, appears comparable between the two strategies, with some evidence even suggesting a potential late survival benefit for TEVAR. These findings necessitate a nuanced interpretation, particularly when applied to a younger patient demographic who have a longer life expectancy and for whom the long-term durability of a prosthetic graft or stent is of paramount importance.

The markedly lower perioperative mortality associated with TEVAR, as demonstrated in both ruptured and intact aneurysm settings across multiple large, nationwide studies [16,18,20], is a robust and expected finding that aligns with the broader literature on aortic repair. This mortality benefit is largely attributed to the minimally invasive nature of TEVAR, which avoids the physiologic insults of a thoracotomy, single-lung ventilation, and aortic cross-clamping. Our review corroborates the findings of major trials and registries in older, higher-risk populations. For instance, the INSTEAD trial demonstrated the safety of TEVAR in subacute dissection, while the EUROSTAR registry established its feasibility with acceptable early mortality [21,22]. The consistency of this signal across diverse healthcare systems and patient cohorts, from the ruptured aneurysm data of Ultee et al. [20] and Yamaguchi et al. [16] to the intact aneurysm analysis of Orelaru et al. [13], reinforces TEVAR as the preferred strategy for mitigating early death, especially in acute presentations, a finding echoed in a large meta-analysis by Cheng et al. [23].

Beyond mortality, the perioperative morbidity profile further favors TEVAR in the immediate post-operative period. The significant reduction in post-operative stroke rates observed by Orelaru et al. [13] is a critical outcome, as neurological injury profoundly impacts quality of life. This is likely due to the avoidance of aortic arch manipulation and cross-clamping, reducing the risk of atheromatous embolization, a mechanism previously described by Ullery et al. [24]. However, the similar rates of SCI between TEVAR and OAR reported in the same study [13] highlight that paraplegia remains a devastating complication common to both procedures, albeit through different mechanisms; OAR risks ischemia during cross-clamp time, while TEVAR risks coverage of critical segmental arteries without adequate revascularization. Furthermore, the substantial reductions in hospital length of stay and transfusion requirements with TEVAR [13,16] are not merely metrics of convenience but are strong proxies for reduced procedural trauma, fewer complications, and more rapid recuperation, leading to significant healthcare economic benefits and earlier return to functional status for younger, often employed, patients.

Despite these compelling short-term advantages, the most striking and consistent finding of this review is the stark contrast in long-term durability, as evidenced by the significantly higher reintervention rates for TEVAR. The studies by Orelaru et al. [13], Muncan et al. [15], and Chiu et al. [19] all report a three- to fivefold increased hazard of reoperation following TEVAR. This is a pivotal concern in younger patients. The reasons are multifactorial. Endoleaks (particularly types Ia, Ib, and III), device migration, stent-graft fatigue, and disease progression in the native aorta are well-documented failure modes of ER. This is especially pertinent in patients with CTDs, like those studied by Muncan et al. [15], whose fragile aortic tissue provides a poor landing zone for stent-grafts, predisposing to complications and necessitating reoperation. Our findings thus resonate with and are strengthened by existing literature, such as the work of Olsson et al. [25], which emphasizes the high failure rate of TEVAR in Marfan syndrome patients, and the long-term follow-up from the VALOR trial [26], which showed a steady accrual of reinterventions over time in a younger cohort. For a young patient, a primary TEVAR may not be a definitive procedure but rather the first step in a lifelong management pathway involving repeated imaging and potential re-interventions, each carrying its own risk and psychological burden.

The comparison of long-term survival between the two strategies reveals a complex and somewhat contradictory picture. The 10-year survival data from Orelaru et al. [13] showed no significant difference, suggesting that the early mortality benefit of TEVAR may be eroded over time, possibly by the consequences of the very reinterventions and late complications it incurs. In contrast, the analysis by Chiu et al. [19] pointed towards a superior mean survival with TEVAR, equivalent to a gain of over 200 days. This discrepancy may be explained by differences in study population, follow-up duration, and statistical methodology. It is plausible that the survival advantage of TEVAR is most pronounced in higher-risk patients, where the perioperative mortality differential is largest, and that over very long periods (e.g., 15-20 years), the curves may converge or even cross. This hypothesis finds some support in the long-term follow-up of the OVER trial for abdominal aortic aneurysms, where an early survival advantage for EVAR was lost after several years [27]. The acceptable five-year survival reported by Lin et al. [14] in a complex cohort requiring open repair after prior TEVAR further illustrates that salvage procedures are feasible, but they represent a high-risk scenario that ideally should be avoided through appropriate initial patient selection.

When contextualizing these findings for the younger patient (<65 years), several critical considerations emerge that extend beyond the raw outcome data. First, the issue of prosthetic durability is magnified. A 40-year-old patient may require 40+ years of service from a stent-graft, a demand far exceeding the current long-term data and the engineered lifespan of these devices. The potential for device fatigue, material failure, and the need for multiple reinterventions over a lifetime present a formidable challenge. Second, the management of CTDs in young adults demands a particularly cautious approach. While Muncan et al. [15] included CTD patients, the current consensus, echoed in guidelines from the European Society for Cardiology and the American Heart Association, strongly favors open repair with graft interposition in CTD patients requiring elective surgery, precisely due to the poor long-term results of TEVAR in this population, a stance strongly supported by the findings of Kieffer et al. [28]. Our review supports this stance, as the high reintervention rate in the TEVAR group [15] underscores its unsuitability as a first-line elective repair in this subset. Third, the patient's life stage must be considered. A younger patient may be less willing to accept the prolonged recovery and initial invasiveness of OAR, prioritizing a quicker return to work and family life, even with the understanding of a higher future reintervention risk. This shared decision-making process must be thoroughly informed by the trade-offs elucidated in this review.

Limitations

This systematic review has several limitations that must be acknowledged. Primarily, the entirety of the evidence base is derived from retrospective, observational studies, which are inherently susceptible to selection bias and unmeasured confounding. For instance, patients selected for OAR may have had more complex anatomy or been younger and fitter, while those selected for TEVAR may have been higher-risk surgical candidates, potentially biasing the outcomes in either direction. While some studies employed propensity score matching [13,15] to mitigate this, residual confounding remains a possibility. The risk of bias assessment, while largely indicating low risk for most studies, identified serious concerns in two key studies [15,19], tempering the strength of certain conclusions. Furthermore, significant clinical heterogeneity was present across the included studies, including variations in the proportion of ruptured vs. intact aneurysms, the inclusion of patients with CTDs, different surgical techniques, and evolving device technology over the long study period. This heterogeneity precluded a meta-analysis and makes direct comparisons challenging. Finally, the reporting of long-term outcomes, particularly patient-centered outcomes like quality of life and aortic-specific morbidity beyond reintervention, was inconsistent and often incomplete.

## Conclusions

For younger patients under 65 years of age with DTAA, the choice between TEVAR and OAR represents a strategic trade-off between superior perioperative safety and long-term durability. TEVAR unequivocally offers lower early mortality and morbidity, fewer strokes, and a faster recovery. However, this comes at the cost of a substantially higher lifetime risk of reintervention. Long-term survival appears similar, though the evidence is not entirely consistent. Therefore, the decision must be individualized, considering factors such as aneurysm etiology (especially the presence of connective tissue disorders), anatomical suitability for TEVAR, surgical risk, and, crucially, the patient's values and preferences regarding short-term recovery versus long-term freedom from reoperation. TEVAR is an invaluable tool, particularly for high-risk acute presentations and patients with favorable anatomy who accept the need for vigilant surveillance. For young, fit patients with CTDs or those seeking a potentially definitive single intervention, open repair remains a cornerstone of therapy. Future prospective, long-term studies and registries focused specifically on this younger demographic are urgently needed to refine these recommendations and guide the optimal application of these complementary techniques.
